# The prevalence and immune response to coinfection by avian haemosporidians in wild Eurasian blackbirds *Turdus merula*

**DOI:** 10.1017/S0031182024000829

**Published:** 2024-11

**Authors:** Ellie Lebeau, Jenny C. Dunn

**Affiliations:** 1 Joseph Banks Laboratories, School of Life and Environmental Sciences, University of Lincoln, Lincoln, UK; 2 School of Biology, University of Leeds, Leeds, UK

**Keywords:** coinfection, *Haemoproteus*, haemosporidian, immune response, *Leucocytozoon*, PCR, *Plasmodium*

## Abstract

Coinfection of a host by more than 1 parasite is more common than single infection in wild environments and can have differing impacts, although coinfections have relatively rarely been quantified. Host immune responses to coinfection can contribute to infection costs but are often harder to predict than those associated with single infection, due to the influence of within-host parasite–parasite interactions on infection virulence. To first quantify coinfection in a common bird species, and then to test for immune-related impacts of coinfection, we investigated the prevalence and immune response to avian haemosporidian (genera: *Plasmodium*, *Haemoproteus* and *Leucocytozoon*) coinfection in wild blackbirds. Coinfection status was diagnosed using a 1-step multiplex polymerase chain reaction, immune response was quantified through white blood cell counts and heterophil: lymphocyte ratios, and parasitaemia was quantified for each infected sample. We detected high rates of haemosporidian infection and coinfection, although neither impacted immune activity, despite a significantly higher parasitaemia in individuals experiencing double *vs* single infection. This suggests that immune-related costs of haemosporidian single and coinfection are low in this system. This could be due to long-term host–parasite coevolution, which has decreased infection virulence, or a consequence of reduced costs associated with chronic infections compared to acute infections. Alternatively, our results may obscure immune-related costs associated with specific combinations of coinfecting haemosporidian genera, species or lineages. Future research should investigate interactions that occur between haemosporidian parasites within hosts, as well as the ways in which these interactions and resulting impacts may vary depending on parasite identity.

## Graphical abstract

**Figure:**
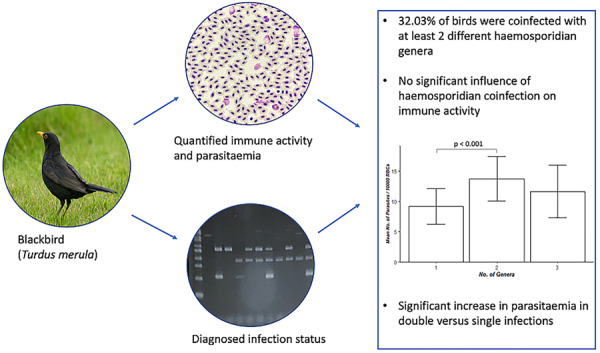

## Introduction

Coinfection refers to the concurrent infection of an individual host with at least 2 genetically distinct parasites (Hoarau *et al*., 2020) and is more common than single infections in natural populations (Telfer *et al*., 2010; McArdle *et al*., 2018). The outcomes of coinfection can differ significantly from that of single infections, as the presence of multiple different parasites alters within-host infection dynamics, thereby influencing disease severity (Gibson *et al*., 2011). Coinfection is often more costly to hosts than single infection (Bordes and Morand, 2011). For example, mice infected with *Plasmodium berghei* and *Trypanosoma brucei* experienced more severe anaemia and greater mortality than singly infected mice (Ademola and Odeniran, 2016). However, coinfections can also be less detrimental than single infections, as evidenced by the increased survival rates of mice infected with 2 strains of *T. brucei brucei* compared to those infected with only 1 (Balmer *et al*., 2009). Improving our ability to accurately predict the impacts of coinfections on wild host individuals would improve our understanding of the evolution and ecology of parasites and hosts, and of the mechanisms by which they may influence populations (Thomas *et al*., 2022). However, most studies that focus on the impacts of parasitic infection still only evaluate the effects of single infections (Thumbi *et al*., 2014). Of the studies that have investigated coinfections, many focus on experimental infections, which may neglect to account for the various factors that influence infection outcomes in wild populations (Hananeh *et al*., 2022).

Measuring the immune response to parasite coinfection in wild hosts could help to gain a deeper understanding of the direct and indirect fitness-related impacts of coinfection on wild host individuals and populations (Budischak *et al*., 2012), particularly as immune responses respond quickly to physiological stress and tend to be more sensitive to changes in host condition than standard body fat indices (Budischak *et al*., 2012). Immune responses are costly to the host, as immune activation and maintenance is energetically expensive (Ots *et al*., 2001) and may lead to autoimmunity (Råberg *et al*., 1998). Immunity is also traded-off against other life-history traits, such as reproduction, which can further reduce fitness by reducing host viability (Baer and Schmid-Hempel, 1999) or parental effort (Råberg *et al*., 2000). The fitness costs of an immune response tend to increase as the response intensifies (Marzal *et al*., 2007). Therefore, although immune responses are predicted to increase in strength as infection virulence increases (de Lope *et al*., 1998), this does not always occur, as hosts may need to suppress immunity, particularly if they are already experiencing stressful conditions (Hanssen *et al*., 2004). Immune responses to coinfection can be even more difficult to predict than immune responses to single infection, due to the influence of parasite–parasite interactions on infection virulence. This is exemplified by the conflicting results obtained from research into the immune response to coinfection. For instance, Vieira-Santos *et al*. (2021) found that mice coinfected with *P. berghei* and *Ascaris suum* had significantly elevated white blood cell (WBC) counts compared to singly infected mice. However, Olifiers *et al*. (2015) detected lower WBC counts in male coatis (*Nasua nasua*) infected with *T. cruzi* and *T. evansi* than in individuals with single infections. This highlights the complexity of coinfection and the need for further investigation into its immune-related impacts.

Avian haemosporidian parasites (genera: *Plasmodium*, *Haemoproteus* and *Leucocytozoon*) can cause avian malaria and malaria-like disease and are frequently used as models in studies of host–parasite dynamics (Lachish *et al*., 2011). Acute infection of the avian host can cause anaemia, lethargy, anorexia and even death (Krams *et al*., 2013; Schoener *et al*., 2014), and chronic infections, although often asymptomatic, can impact host life history traits (Knowles *et al*., 2010). Research into the immune response to avian malaria infection has generated contradictory results. Some studies have reported increased WBC counts in response to haemosporidian infection (e.g. Wojczulanis-Jakubas *et al*., 2012; Ellis *et al*., 2015), whereas others have detected no difference in haematological parameters (Krams *et al*., 2013; Vanstreels *et al*., 2019). This variability could in part be attributed to a high prevalence of avian haemosporidian coinfection (Pigeault *et al*., 2018). Across the 3 genera of avian malaria and malaria-like parasites, there are over 248 species and 5121 lineages (MalAvi Database, accessed 10/05/2024; Bensch *et al*., 2009). Coinfection with multiple of these genera, species or lineages is common in wild birds, for example, of the 54 infected Great tits (*Parus major*) sampled by Rooyen *et al*. (2013), 81.5% were coinfected with at least 2 different haemosporidian genera. Coinfection with multiple different haemosporidian parasites can significantly impact host individuals and has been reported as being more virulent than single infection (Palinauskas *et al*., 2011). Therefore, the immune response to avian malaria coinfection could be expected to be greater than that associated with single infections. However, the complexity of within-host parasite–parasite interactions coupled with the minimal amount of research on this topic makes it difficult to form such general conclusions.

Here, we investigate the immune response to coinfection by multiple genera of avian Haemosporidia in a species of wild bird known to have a high prevalence of blood parasites, and thus predicted to have a high prevalence of coinfection. We predict that (1) immune responses will be stronger in singly infected birds than uninfected birds, as single infection can exert significant costs on hosts (Knowles *et al*., 2010). We also hypothesize that (2) coinfected birds will exhibit stronger immune responses than singly infected birds, as avian malaria coinfection has been reported as more virulent than single infection (Palinauskas *et al*., 2011), and immune responses are expected to increase in intensity where there is potential for greater damage to the host (de Lope *et al*., 1998).

## Materials and methods

### Blood samples

A total of 128 blood samples were examined during this study, all of which had been previously collected from blackbirds (*Turdus merula*) at the following sites in Lincolnshire, UK, between 2017 and 2022: North Carlton (53°17′N, 0°34′W), Eagle (53°11′N, 0°41′W), Owmby (53°31′N, 0°22′W), Blankney (53°07′N, 0°24′W), Moorlands (53°12′N, 0°34′W) and Industrial Cottages (53°14′N, 0°33′W). Two blood smears were created from each sample, which were fixed in absolute methanol and stained with Giemsa for 45–50 min following standard protocols. Additional blood was stored at −20°C prior to DNA extraction and subsequent analysis.

### DNA extraction and determination of infection status

DNA was extracted from all samples using a DNeasy Blood and Tissue Kit (Qiagen, Hilden, Germany) according to the manufacturer's instructions. The infection status of each sample was determined using a 1-step multiplex polymerase chain reaction (PCR) assay capable of detecting parasites of the genera *Plasmodium* and *Leucocytozoon* as well as the subgenera *Haemoproteus* and *Parahaemoproteus* (genus: *Haemoproteus*) (Ciloglu *et al*., 2023). However, we did not expect to find *Haemoproteus* parasites in our samples as this subgenus tends to infect birds from the orders Suliformes, Charadriiformes and Columbiformes, rather than Passeriformes (Ciloglu *et al*., 2023). The PCR was set up in total volumes of 10 μL, consisting of 5 μL of commercial multiplex PCR master mix (2 × Qiagen Multiplex PCR Master Mix, Qiagen), 0.2 μL of each primer (10 mm concentration) (Table 1), 2.4 μL of ddH_2_O and 1 μL of DNA. Reactions were performed in a Bio-Rad T100 Thermal Cycler (Bio-Rad Laboratories, Hercules, CA, USA). The PCR protocol began with denaturation at 95°C for 15 min, after which there were 37 cycles of 94°C for 30 s, 58.9°C for 90 s and 72°C for 30 s, and finally terminal extension at 72°C for 10 min. One positive and one negative control were included for every 10 samples, to ensure DNA had been successfully amplified (positive control) and that no contamination was present (negative control). Positive controls contained 1 μL of DNA from a blood sample confirmed to be naturally infected with *Plasmodium*, *Leucocytozoon* and *Parahaemoproteus* parasites, whereas negative controls contained 1 μL of ddH_2_O in place of DNA.

**Table 1.** Names and sequences of all primers used in the 1-step multiplex PCR and *Leucocytozoon* sequencing PCR, along with product sizes

Parasite taxa	Primer name	Primer sequence (5′ to 3′)	Product size (bp)	Reference
One-step multiplex PCR				
*Plasmodium*	PMF	CCTCACGAGTCGATCAGG	~378	Ciloglu *et al*. (2019)
	PMR	GGAAACCGGCGCTAC		
*Leucocytozoon*	LMF	TGGAACAATAATTGSATTATTTACAYT	~218	Ciloglu *et al*. (2019)
	LMR	AACATATCATATTCCATCCATTTAGATTA		
Subgenus *Parahaemoproteus*	HMF	ATTGGATGTCAATTACCACAATC	~533	Ciloglu *et al*. (2019)
	HMR	GGGAAGTTTATCCAGGAAGTT		
Subgenus *Haemoproteus*	HHMF	ATTTCTACAAAATGCCAGTATAATAATAC	~471	Ciloglu *et al*. (2019)
	HHMR	CTGGGGTATTTTACATTTTGCAC		
*Leucocytozoon* sequencing PCR				
*Leucocytozoon*	Leunew1F	GGWCAAATGAGTTTCTGGG	~340	Quillfeldt *et al*. (2014)
	LDRd	CTGGATGWGATAATGGWGCA		Merino *et al*. (2008)

Amplification products (5 μL) were electrophoretically resolved after 1 h at 120 V in 3% agarose gels. Gels were then post-stained with gel red to prevent the dye from potentially interfering with DNA migration during electrophoresis. During post-staining, gels were placed in a plastic tub containing 100 ml of water and 30 μL of gel red, which was gently agitated for 45 min. After this, gel visualization was carried out using a Syngene InGenius 3 transilluminator (Syngene International, Bengaluru, Karnataka) and GeneSys software (Genesys, Menlo Park, CA, USA). The presence of an amplicon band at the expected size (Table 1) was indicative of infection. Samples were recorded as either uninfected, singly infected or coinfected with parasites belonging to multiple different genera and/or subgenera, and the identity of any parasites present was noted.

### Sequence analysis

Positive samples were sent to Macrogen Europe (Amsterdam, Netherlands) to be sequenced, in order to confirm the identity of the parasite strains present. Although the above PCR protocol produced sufficient lengths of *Plasmodium* and *Parahaemoproteus* DNA to allow for meaningful sequencing, this was not the case for *Leucocytozoon* DNA. As such, *Leucocytozoon* DNA was amplified using Leunew1F and LDRd primers (Table 1) prior to sequencing, which produced a product of length 340 bp. The PCR protocol was as follows: 95°C for 15 min, followed by 34 cycles of 95°C for 30 s, 56°C for 30 s and 72°C for 1 min, and finally terminal extension at 72°C for 10 min. This PCR was carried out using the same equipment and reagents as detailed above. Reaction volumes for all reagents were also identical to those mentioned previously, apart from that of ddH_2_O (3.6 μL in every 10 μL reaction).

Forward sequences were assessed for any errors, trimmed and then aligned using AliView (Larsson, 2014). Sequences of poor quality, where double peaks were present throughout the chromatogram, were not included in any further analysis. *Leucocytozoon* sequences were queried using the BLAST algorithm for both the MalAvi (Bensch *et al*., 2009) and GenBank databases to identify the closest matching sequences. *Plasmodium* and *Parahaemoproteus* sequences did not overlap the MalAvi region and were therefore only queried against the GenBank database.

### Immune activity and infection intensity

Blood smears were assessed under oil immersion at 100× magnification to quantify immune responses and infection intensity. Inspection of each slide was concluded after a total of both 100 WBCs and 10 000 red blood cells (RBCs) had been examined. Each WBC inspected was identified based on standard avian guidelines (Clark *et al*., 2009). Immune function was then quantified through calculation of standardized WBC counts and heterophil to lymphocyte ratios (H:L ratios), which are used as measures of immune activity (Smits, 2007) and chronic stress (Davis *et al*., 2008), respectively. WBC counts were calculated as the number of erythrocytes present for every 100 WBCs and H:L ratio was calculated using the following formula: heterophils/(heterophils + lymphocytes) (Dunn *et al*., 2017). Infection intensity was measured for each blood smear by counting the number of parasites (all genera) present for every 10 000 RBCs.

### Statistical analysis

All statistical analyses were carried out in R version 4.1.1 for Windows (R Core Team, 2021). General linear models with Gaussian error structure were used to examine the relationship between immune activity and infection status. H:L ratio was square root transformed and WBC count log-transformed to achieve normal distributions. To examine the relationship between coinfection status (coinfected or not coinfected) and immune function, models were run with either H:L ratio or WBC count as the dependent variable and coinfection status as a fixed factor. The effect of genera number (0, 1, 2 or 3, a categorical variable) on immune activity was assessed using models run as above but with genera number as a fixed factor. As parasitaemia is known to influence immune activity (Sol *et al*., 2003; Ellis *et al*., 2015), this was included as a covariate in the above models. The effect of genera number (0, 1, 2 or 3) on parasitaemia was investigated using a Poisson regression model constructed with parasitaemia as the dependent variable and genera number as a fixed factor. Likelihood ratio tests were used to test the significance of model terms. Sample sizes were too small to facilitate investigation of the effects of infection with specific parasite genera or genus combinations.

### Co-occurrence analysis

To test whether coinfecting genera were significantly associated with one another, or whether coinfections occurred at random, we used the R co-occur package (Griffith *et al*., 2016) to test whether the observed frequency of genus co-occurrence is greater or less than expected given the overall prevalence of each genus in the population.

## Results

### Haemosporidia prevalence

**Figure 1.**
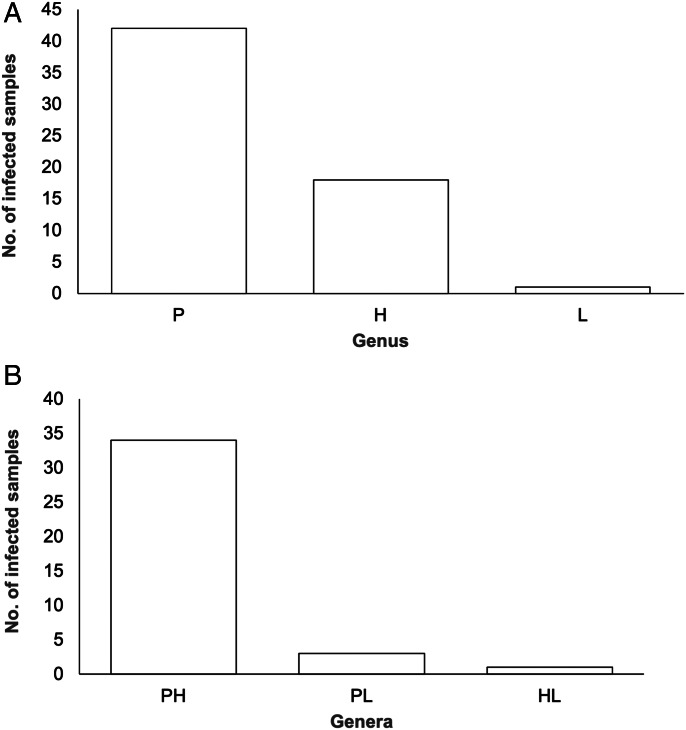
Number of samples (a) singly infected and (b) doubly infected with parasites belonging to each genus/genera combination.

A total of 128 blood samples were examined, of which 26 were uninfected (20.31%), 61 were singly infected (47.66%) and 41 were coinfected with at least 2 different haemosporidian genera (32.03%). Of these 41 samples, 38 were infected with 2 genera (92.68%) and 3 contained all 3 genera (7.32%). Singly infected samples were most commonly infected by *Plasmodium* spp. (42), followed by *Parahaemoproteus* spp. (18) and then *Leucocytozoon* spp. (1) (Fig. 1a). *Plasmodium* spp.*–Parahaemoproteus* spp. infections accounted for 89.47% (34) of double infections, whereas *Plasmodium* spp.–*Leucocytozoon* spp. and *Leucocytozoon* spp.–*Parahaemoproteus* spp. infections were much less common, making up 7.89% (3) and 2.63% (1) of double infections respectively (Fig. 1b). No samples were infected with the subgenus *Haemoproteus*, and 1 sample infected with *Plasmodium* spp. was also infected with microfilaria (identified during microscopy). The mean infection intensity amongst birds confirmed as infected through PCR was 8.75 parasites per 10 000 erythrocytes (range 0–163 parasites per 10 000 erythrocytes). In 12 singly infected and 3 coinfected samples identified using PCR, no parasites were seen under the microscope (10 *Plasmodium* spp.-infected, 2 *Parahaemoproteus* spp.-infected and 3 infected with *Plasmodium* spp. and *Parahaemoproteus* spp.). Three samples tested negative for *Leucocytozoon* spp. and 2 tested negative for *Parahaemoproteus* spp. using PCR, despite parasites being identified on blood smears. Photographs of each parasite genus seen during microscopic examination can be found in Supplementary file 1.

### Sequence analysis

We were able to obtain 54 good quality sequences, of which 17 were identified as *Plasmodium* spp., 32 were *Parahaemoproteus* spp. and 5 were *Leucocytozoon* spp. We identified 5 unique *Plasmodium* spp. sequences, 11 unique *Parahaemoproteus* spp. sequences, and 4 unique *Leucocytozoon* spp. sequences (Table 2). Eleven of the 54 sequences were obtained from coinfected samples (1 sample was infected with Plas1, Haem1 and Leuc1 concurrently, 1 with Haem10 and Leuc2, 1 with Haem5 and Plas3, and 2 samples were infected with Plas1 and Haem1).

**Table 2.** Haemosporidian lineages sequenced from infected samples as part of this study, alongside their closest matches on MalAvi and GenBank

Lineage (this study)	Parasite genus	MalAvi match	% identity	GenBank match	% identity	*N*	Citation
Plas1	*Plasmodium*	NA	NA	KY653813.1 KY653792.1	100	8	Pacheco *et al*. (2018)
Plas2	*Plasmodium*	NA	NA	MG598392.1 KY653815.1 KY653802.1 KY653785.1 KY653780.1 KY653766.1 OP701684.1 KC138226.1 NC_008279.1 AY733089.1	99.67	4	Ferreira-Junior *et al*. (2018) Pacheco *et al*. (2018) Lotta-Arévalo *et al*. (2023) Mantilla *et al*. (2013) Omori *et al*. (2007) Beadell and Fleischer (2005)
Plas3	*Plasmodium*	NA	NA	KY653749.1 KY653814.1 KY653813.1 KY653804.1 KY653792.1 KY653786.1 KY653771.1 KY653770.1 AB599930.1 NC_008288.1	97.31	2	Pacheco *et al*. (2019) Pacheco *et al*. (2018) Hikosaka *et al*. (2011) Omori *et al*. (2007)
Plas4	*Plasmodium*	NA	NA	KY653762.1	100	2	Pacheco *et al*. (2018)
Plas5	*Plasmodium*	NA	NA	MG598392.1 KY653815.1 KY653804.1 KY653802.1 KY653786.1 KY653785.1 KY653780.1 KY653766.1 OR347675.1 OR347674.1 OP701684.1 KC138226.1 AB599930.1 NC_008288.1 NC_008279.1 AY733089.1	99.31	1	Ferreira-Junior *et al*. (2018) Pacheco *et al*. (2018) Musa (unpublished data) Böhme *et al*. (2018) Lotta-Arévalo *et al*. (2023) Mantilla *et al*. (2013) Hikosaka *et al*. (2011) Omori *et al*. (2007) Beadell and Fleischer (2005)
Leuc1	*Leucocytozoon*	TUMER01	100	KJ488790.1 KJ488693.1	100	2	Drovetski *et al*. (2014)
Leuc2	*Leucocytozoon*	TUMER01 TUMER07	100	OQ311222.1 OQ311218.1 OQ311186.1 OQ311165.1 OQ311161.1 OQ311159.1 OQ311146.1 OQ311097.1 OQ311093.1 OQ311090.1 OQ311058.1 OQ311009.1 ON730885.1 KJ488828.1 KJ488790.1 KJ488693.1 GU391353.1	100	1	Strehmann *et al*. (2023) Díez-Fernández *et al*. (2023) Drovetski *et al*. (2014) Hellgren *et al*. (unpublished data)
Leuc3	*Leucocytozoon*	TUMER19	95	KJ488895.1	94.67	1	Drovetski *et al*. (2014)
Leuc4	*Leucocytozoon*	COLBF08	99	KR052940.1	99.23	1	Murdock *et al*. (2015)
Haem1	*Haemoproteus*	NA	NA	KY653763.1	100	19	Pacheco *et al*. (2018)
Haem2	*Haemoproteus*	NA	NA	KY653763.1	97.74	2	Pacheco *et al*. (2018)
Haem3	*Haemoproteus*	NA	NA	KY653763.1	99.33	2	Pacheco *et al*. (2018)
Haem4	*Haemoproteus*	NA	NA	KY653763.1	99.56	1	Pacheco *et al*. (2018)
Haem5	*Haemoproteus*	NA	NA	KY653763.1	99.55	1	Pacheco *et al*. (2018)
Haem6	*Haemoproteus*	NA	NA	KY653763.1	98.63	2	Pacheco *et al*. (2018)
Haem7	*Haemoproteus*	NA	NA	KY653763.1	98.89	1	Pacheco *et al*. (2018)
Haem8	*Haemoproteus*	NA	NA	KY653763.1	99.13	1	Pacheco *et al*. (2018)
Haem9	*Haemoproteus*	NA	NA	KY653763.1	98.41	1	Pacheco *et al*. (2018)
Haem10	*Haemoproteus*	NA	NA	KY653763.1	99.29	1	Pacheco *et al*. (2018)
Haem11	*Haemoproteus*	NA	NA	KY653763.1	99.31	1	Pacheco *et al*. (2018)

There are no data for *Plasmodium* and *Haemoproteus* MalAvi matches as the amplified DNA region does not overlap with the MalAvi region.

Plas1 was isolated from 8 individuals within this study and was identical to sequences of both *Plasmodium vaughani* collected from a blackbird in Lithuania and *Plasmodium unalis* collected from a Great thrush (*Turdus fuscater*) in Colombia (Table 2). Plas2 was isolated from 4 individuals and was a new sequence with 99.67% similarity to 10 sequences, detailed in Table 2. Plas3 was isolated from 2 blackbirds in this study, and was a 97.31% match to 10 sequences on GenBank: *Plasmodium juxtanucleare* from a short-crested flycatcher (*Myiarchus ferox*) in Brazil, 2 sequences of *P. unalis* from Great thrushes in Colombia, *P. vaughani* from a blackbird in Lithuania, *Plasmodium homopolare* from a rufous-collared sparrow (*Zonotrichia capensis*) in Colombia, 3 sequences of *Plasmodium gallinaceum* from a domestic chicken (*Gallus gallus domesticus*) and *Plasmodium* sp. from Swainson's thrush (*Catharus ustulatus*) in Colombia, an African penguin (*Spheniscus demersus*) in South Africa, and a Northern bobwhite quail (*Colinus virginianus ridgwayi*) in the USA. Plas4 was found in 2 blackbirds in this study, and was identical to only 1 sequence, *Plasmodium circumflexum*, isolated from a wren (*Troglodytes troglodytes*) in Lithuania. We isolated Plas5 from 1 blackbird: this was a new sequence with 99.31% identity to 17 sequences in GenBank, detailed in Table 2.

Haem1, isolated from 19 blackbirds in this study, was identical to 1 sequence on GenBank, *Haemoproteus minutus*, isolated from a blackbird in Lithuania. Haem2–Haem11 were all new sequences, with 97–99% similarity to the same sequence as Haem 1 (Table 2).

Leuc1, isolated from 2 blackbirds in this study, was identical to TUMER01 at the region of overlap with the MalAvi region (235 bp). This lineage has been reported mostly from blackbirds across western Europe and North Africa, with 1 report from a house sparrow (*Passer domesticus*) in the Azores. Leuc2, isolated from 1 individual, was identical to both TUMER01 and TUMER07 at the region of overlap; TUMER07 has only previously been reported from a single blackbird in Portugal. Leuc3, also isolated from a single blackbird, may be a new lineage, with 95% match to TUMER19 on MalAvi at the region of overlap: TUMER19 was isolated from a blackbird in Armenia. Leuc4 was a 99% match at the MalAvi region and a 99.23% match on GenBank to lineage COLBF08 isolated from the Woodland Black Fly (*Simulium silvestre*) in the USA: this lineage had not previously been isolated from an avian host (Table 2).

### Impacts of infection

**Figure 2.**
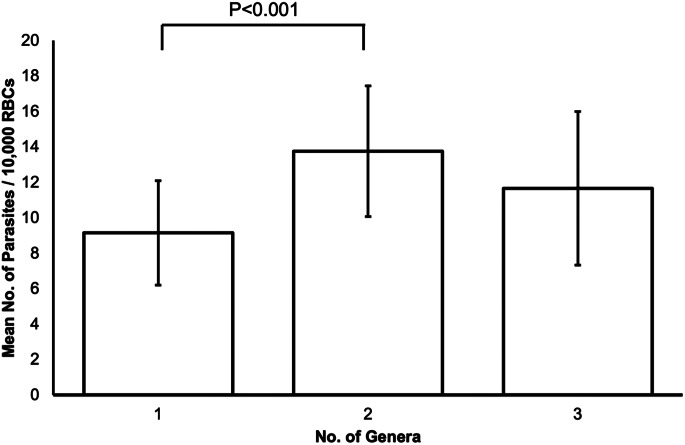
Mean number of parasites per 10 000 red blood cells in blackbird (*Turdus merula*) blood smears infected with 1, 2 or 3 avian haemosporidian genera (singly infected: *n* = 61; 2 genera: *n* = 38; 3 genera: *n* = 3). Error bars represent ±1s.e.

No significant differences in H:L ratio (*F*_1_ = 0.27, *P* = 0.60) or WBC count (*F*_1_ = 0.78, *P* = 0.38) were detected between coinfected blackbirds (*T. merula*) and those that were not coinfected (H:L ratio, coinfected: 0.199 ± 0.017; not coinfected: 0.185 ± 0.011; WBC count, coinfected: 14 806.767 ± 1663.892; not coinfected: 16 823.315 ± 1280.073). Similarly, haemosporidian genus number (0, 1, 2, 3) also had no significant effect on H:L ratio (*F*_1_ = 0.09, *P* = 0.76; 0:0.190 ± 0.021; 1:0.182 ± 0.013; 2:0.198 ± 0.017; 3:0.210 ± 0.080) or WBC count (*F*_1_ = 1.81, *P* = 0.15; 0 genera: 22 066.158 ± 3421.956; 1:14 588.661 ± 995.793; 2:15 226.516 ± 1774.760; 3:9489.941 ± 1840.128), although there were significant differences in parasitaemia between individuals infected with 1 genus and those infected with 2 genera (χ^2^_2_ = 44.34, *P* < 0.001) (Fig. 2). When controlling for coinfection status, parasitaemia had no significant effect on H:L ratio (*F*_1_ = 0.12, *P* = 0.73) or WBC count (*F*_1_ = 2.20, *P* = 0.14). This was also true when controlling for genus number (H:L ratio: *F*_1_ = 0.13, *P* = 0.72; WBC count: *F*_1_ = 1.36, *P* = 0.25).

### Co-occurrence analysis

At the genus level, associations between genera did not differ from random, given the prevalence of each in the population (*P* > 0.05 for each association). However, all but 1 *Leucocytozoon* spp. infection (*n* = 8) occurred alongside either a *Plasmodium* spp. or a *Haemoproteus* spp. infection.

## Discussion

A high prevalence of avian haemosporidian infection was found amongst sampled blackbirds (*T. merula*), as just under 80% were infected with at least 1 genus of parasite. Although we only sampled birds from 1 area of the UK, other studies have reported high levels of haemosporidian infection in passerine birds from other locations across the UK and Europe (e.g. Hatchwell *et al*., 2000; Valkiūnas *et al*., 2003), suggesting that our findings may be reflective of general infection patterns in this species. We found *Plasmodium* parasites to be the most common of all 3 haemosporidian genera, followed by *Haemoproteus* spp. and then *Leucocytozoon* spp., which concurs with the high incidences of *Plasmodium* spp. reported in blackbirds in various European countries (Dinhopl *et al*., 2015; Himmel *et al*., 2020).

We identified 5 strains of *Plasmodium* spp., 11 strains of *Haemoproteus* spp. and 4 strains of *Leucocytozoon* spp. in sampled blackbirds. Plas1 matched sequences for *P. unalis* and *P. vaughani*. However, as *P. unalis* has been isolated almost exclusively from birds in America (Harl *et al*., 2020), whereas *P. vaughani* is commonly found in Europe, we expect that our samples were infected with *P. vaughani*. As Plas2, Plas3 and Plas5 matched with a considerable number of sequences on GenBank, it is difficult to determine the exact identity of these sequences. However, this suggests that the primers PMF and PMR may be useful in the amplification of a wide variety of *Plasmodium* strains, although further work is needed to determine whether this region may be useful as a barcode for species discrimination. Leuc2 matched with sequences for TUMER01 and TUMER07, although the individual sampled in the present study was more likely infected with TUMER01, given its considerably higher prevalence in this host species.

We also detected a considerable prevalence of haemosporidian coinfection, as 32.03% of all individuals tested positive for infection with at least 2 genera. Similar rates of avian malaria coinfection have been reported in other host species, including a 31% prevalence in American crow nestlings (Townsend *et al*., 2018). These results may be unsurprising considering the high prevalence and diversity of avian malaria parasites globally, and the observation that birds already infected with either *Plasmodium* spp., *Haemoproteus* spp. or *Leucocytozoon* spp. appear more likely to be subsequently infected with another haemosporidian genus (Norte *et al*., 2009). However, there is substantial variation in reported rates of haemosporidian coinfection, for example, coinfection rates recorded by Starkloff and Galen (2023) ranged from 7 to 75% depending on the host population. This is likely due to the many different factors known to influence haemosporidian infection prevalence in the wild, including host species (Lutz *et al*., 2015), migratory behaviour (Ciloglu *et al*., 2020), environmental conditions and the abundance and activity of vectors (Thomas *et al*., 2022).

Contrary to our hypothesis, we detected no significant differences in H:L ratio or WBC count between uninfected and singly infected individuals, suggesting that infection with 1 haemosporidian genus does not impose significant immune-related costs on hosts. This is supported by several studies, all detecting no effect of parasite presence on immunological parameters such as H:L ratio (Krams *et al*., 2013), haematocrit levels (Bichet *et al*., 2020) and WBC count (Vanstreels *et al*., 2019). These results could be a consequence of long-term coevolution between haemosporidian parasites and their avian hosts (Norte *et al*., 2009), which can reduce parasite virulence, subsequently evoking weaker immune responses (Macintosh and Frias, 2017). These coevolutionary relationships can have severe implications for naïve populations, potentially causing high morbidity and mortality (Garnick, 1992; Woodworth *et al.*, 2005). As such, further investigation into the dynamics of coevolutionary relationships should be a priority. However, coevolution has not eliminated all the costs that avian malaria parasites impose upon host individuals, as significant immune responses to these parasites, including increased WBC counts and H:L ratios (Wojczulanis-Jakubas *et al*., 2012; Dunn *et al*., 2013; Ellis *et al*., 2014, 2015), have been recorded in species that are frequently infected by haemosporidians. This evidences the complexities of host–parasite coevolution and the many different factors that can influence immune responses to parasitic infection.

We also found no effect of coinfection on H:L ratio or WBC count. Similar findings were reported by Palinauskas *et al*. (2018), who found no significant differences in body weight between singly infected domestic canaries (*Canaria domestica*) and those infected with 2 different *Plasmodium* species. Likewise, Chavarría *et al*. (2023) found no effect of coinfection on body condition or polychromatophil count in the ash-breasted Sierra finch (*Geospizopsis plebejus*). Conversely, other studies have detected additive costs of coinfection in comparison to single infection, in terms of body condition (Marzal *et al*., 2008) and survival probability (Pigeault *et al*., 2018). The impacts of coinfection on host individuals are dependent on the nature of the interactions occurring between parasites within the host (Karvonen *et al*., 2019). If interactions are antagonistic, infection virulence will be lowered in comparison to single infection, due to the suppression of 1 parasite by another (Clay and Rudolf, 2019; Shen *et al*., 2019). Facultative interactions on the other hand, in which both parasites benefit, can impose greater costs on hosts (Clay and Rudolf, 2019). To fully understand the reasons for the observed differences in coinfection costs, exploration into the interactions that occur between different haemosporidian parasites within avian hosts is needed.

It would be interesting for future work to test for the impacts of coinfection specific to certain parasite species or lineages. This is because different haemosporidian genera, species and lineages exhibit different life history traits, patterns of development inside the avian host and levels of pathogenicity (Atkinson and Riper, 1991; Zehtindjiev *et al*., 2008). For example, Townsend *et al*. (2018) found that, of the 3 avian haemosporidian genera, only *Plasmodium* spp. infection was associated with significant reductions in body condition and haematocrit levels. Additionally, Zehtindjiev *et al*. (2008) reported lower parasitaemia in great reed warblers (*Acrocephalus arundinaceus*) infected with *Plasmodium relictum* compared to individuals infected with *Plasmodium ashfordi*, and Bentz *et al*. (2006) found that parasitaemia of *Plasmodium* lineage TM1 was 100–1000× that of *Plasmodium* TM2 in blackbirds (*T. merula*). These findings suggest that there may also be differences in the ways in which different parasitic genera, species and lineages interact with each other, and with the host, during coinfection (Rooyen *et al*., 2013). Consequently, the immune-related impacts of parasitic coinfections may be dependent on the identity of the parasites present, meaning our results could obscure significant costs associated with specific combinations of haemosporidian parasites. As such, future research should seek to uncover any potential parasite-specific costs of coinfection.

It is also important to acknowledge the impact that the incorrect diagnosis of infection status may have had on our results, as we recorded infection status based solely on the results of the 1-step multiplex PCR, which appears to have failed to detect infection in a few samples. This study also included blood samples that were diagnosed as infected by PCR, but in which no parasites were seen under the microscope. There are a few possible reasons for such observations, including that these birds were suffering from very light parasitaemia, or that parasite DNA detected during PCR represented parasite remnants that had aborted development (Valkiūnas *et al*., 2009; Chagas *et al*., 2016). Consequently, some individuals we recorded as infected might not have been harbouring successful infections. As such, the diagnosis of infection status is probably best achieved using a combination of microscopy and PCR-based techniques.

As well as investigating the immune-related impacts of single infections and coinfections, we also looked at the effect of coinfection on parasitaemia. We found that individuals infected with 2 haemosporidian genera had significantly higher parasitaemia than those infected by 1 genus. As only 3 of our samples were infected with *Plasmodium* spp., *Haemoproteus* spp. and *Leucocytozoon* spp. concurrently, it is possible that this small sample size hampered our ability to also detect a significant influence of 3-genera coinfection on parasitic burden. Other studies have also detected an increase in parasitaemia during coinfections in comparison to single infections (e.g. Palinauskas *et al*., 2018; Chavarría *et al*., 2023), indicating an additive effect of coinfection on parasitaemia (Cox, 2001). However, as we did not record the genus of each parasite seen during microscopic examination, we do not have any data on the relative parasitaemia of each genus in coinfected samples. This restricts our ability to comment on potential dynamics that may have been regulating the parasitaemia of each genus. Previous research has detected both positive (Zehtindjiev *et al*., 2008; Palinauskas *et al*., 2011) and negative relationships (Palinauskas *et al*., 2011) between the parasitaemia of different coinfecting haemosporidians, as well as significant variation in parasitaemia dynamics across coinfected host species. As there is much research demonstrating the significant influence that haemosporidian parasitaemia has on infection costs (e.g. Sol *et al*., 2003; Ellis *et al*., 2015), it is important that the mechanisms that regulate these observed dynamics are investigated further and any changes in these dynamics over the course of infection are recorded.

Contrary to the aforementioned research, we found that parasitaemia had no influence on H:L ratio or WBC count. This could be explained by the generally low levels of parasitic burden detected amongst sampled individuals, suggesting that most birds were suffering from chronic rather than acute infection. As the acute phase of haemosporidian infection imposes greater costs on avian hosts than chronic infection (Krams *et al*., 2013), it is possible that this prevented us from detecting an effect of parasitaemia and infection status on immune activity. Unfortunately, it is more difficult to collect samples from wild birds experiencing acute infection, as these individuals are more likely to reduce their levels of activity and may die before being sampled (Yorinks and Atkinson, 2000; Krams *et al*., 2013). On the other hand, studies that have used anti-Haemosporidia medications to treat chronic infections have uncovered associations between chronic infection and reduced host survival and reproductive success (Knowles *et al*., 2010; Martínez-de la Puente *et al*., 2010). This suggests that even low parasitic burdens can exert significant costs in some circumstances. However, the impacts of haemosporidian infections on wild hosts are dependent on a variety of factors aside from parasitaemia, including food availability and environmental conditions. For example, Cornet *et al*. (2014) found that nutritionally supplemented domestic canaries (*Serinus canaria*) were better able to control *Plasmodium relictum* burdens than those that were not supplemented. It is difficult to assess the extent to which these factors influenced the results of this study, as with any study of haemosporidian infection costs conducted on wild birds. Further examination of the effect of these variables in controlled conditions may help to shed more light on their possible effects in wild environments.

In conclusion, we detected high rates of haemosporidian infection and coinfection, but found no evidence that either impacted immune activity, despite a significantly higher parasitic burden in individuals experiencing double *vs* single infection. This could be a result of coevolution, which has reduced parasite virulence, or a reflection of the reduced costs associated with chronic infection in comparison to acute infection. These findings may obscure significant impacts associated with specific combinations of coinfecting haemosporidian genera, species or lineages. Further research into the interactions that occur between parasites within the host, and how these interactions and resulting impacts may vary depending on parasite identity, would help to facilitate a better understanding of the costs of coinfection in this system.

## Supporting information

Lebeau and Dunn supplementary material Lebeau and Dunn supplementary material

## Data Availability

Sequences generated in this research are accessible through GenBank accession numbers PP850198–PP850217.

## Supplementary material

The supplementary material for this article can be found at https://doi.org/10.1017/S0031182024000829.
