# Supplementary material for: The prevalence and immune response to coinfection by avian haemosporidians in wild Eurasian blackbirds Turdus merula
Source: Parasitology. 2025 Jan 24;151(13):1406–15. doi: 10.1017/S0031182024000829 (PMC12052428; doi:10.1017/S0031182024000829)
Supplement: Lebeau and Dunn supplementary material [file S0031182024000829sup001.docx]

**Supplementary file 1: Photographs of parasites found during microscopic examination.**

**
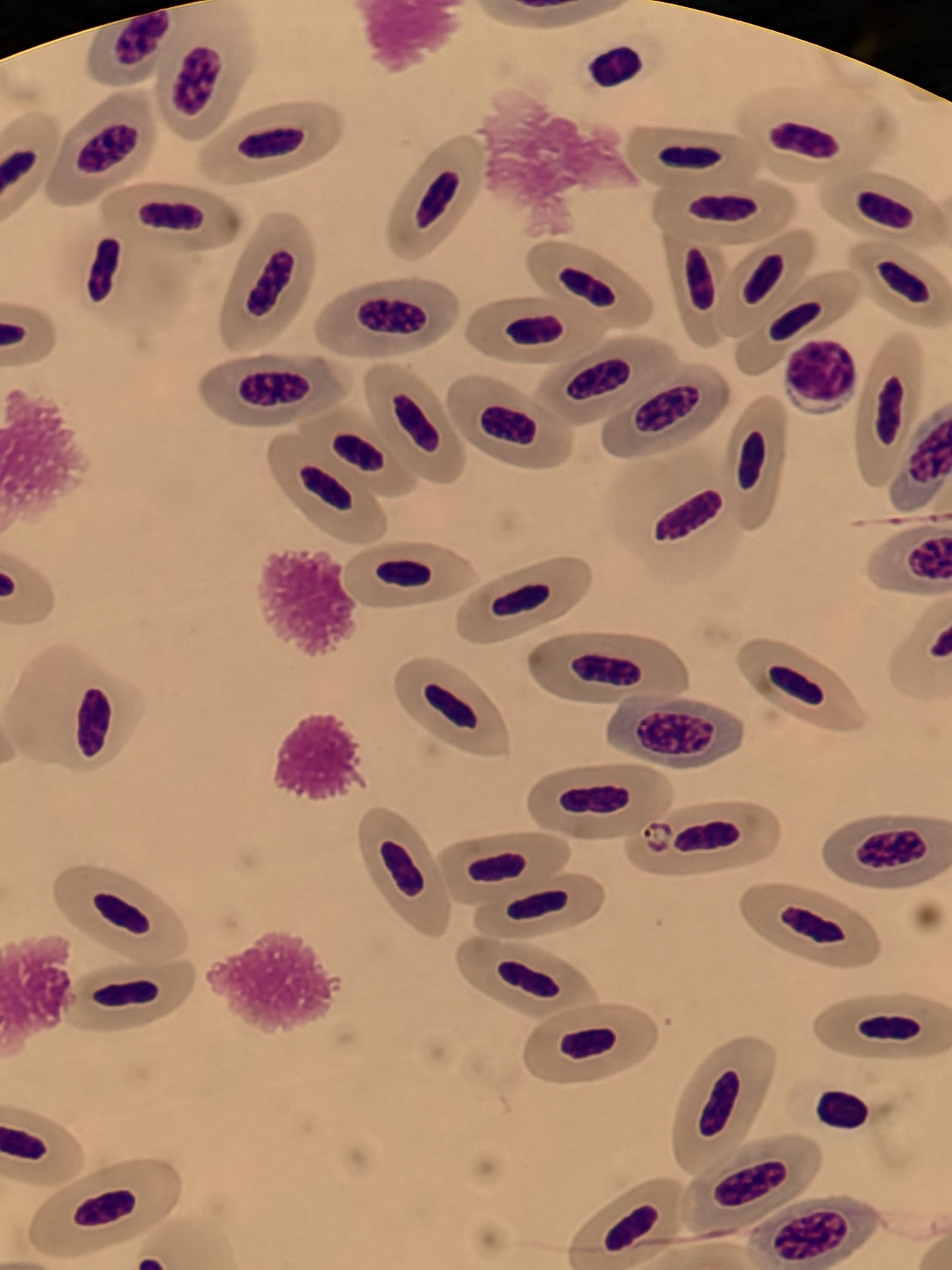
**

***Plasmodium* parasite**

**
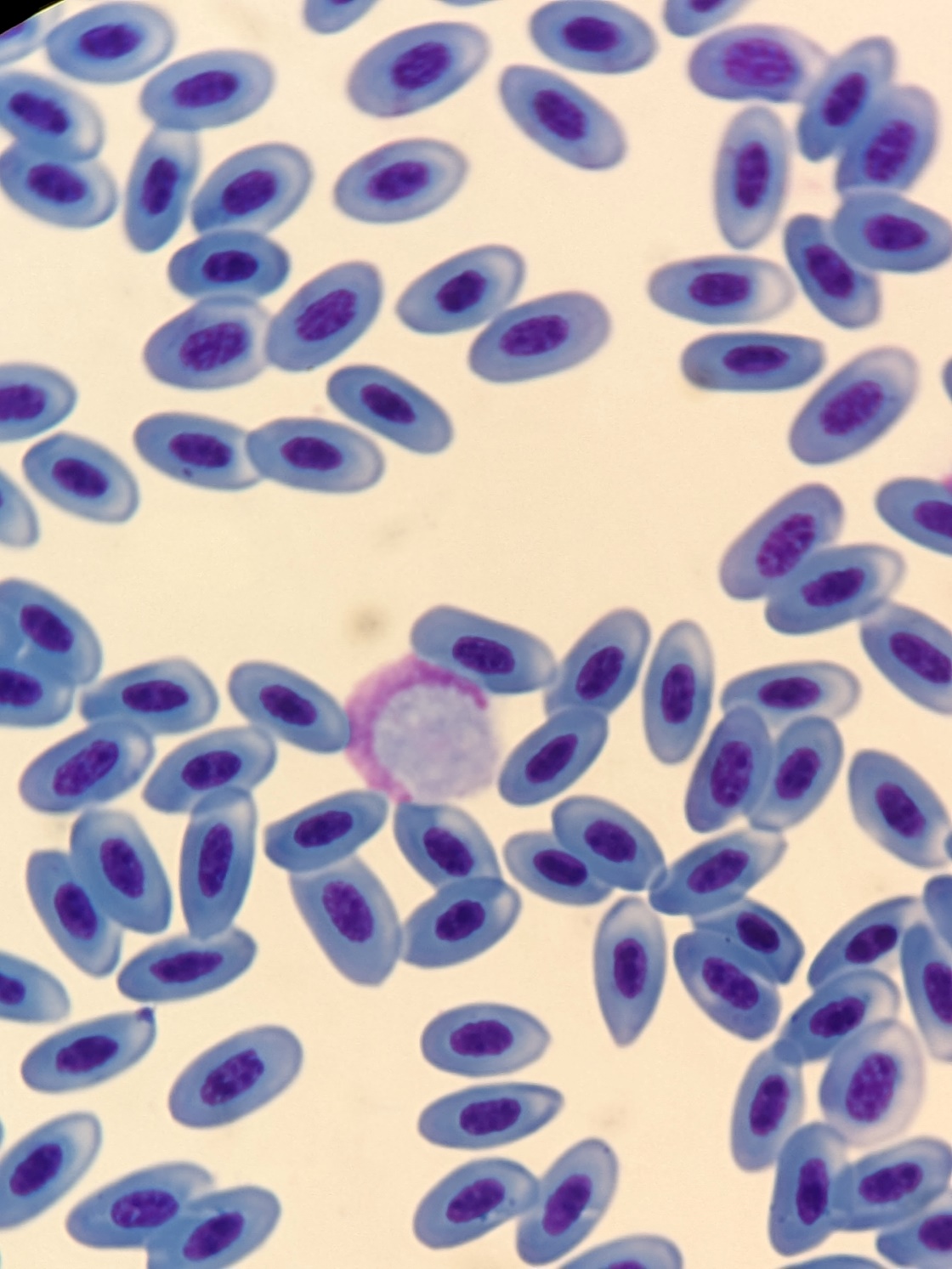

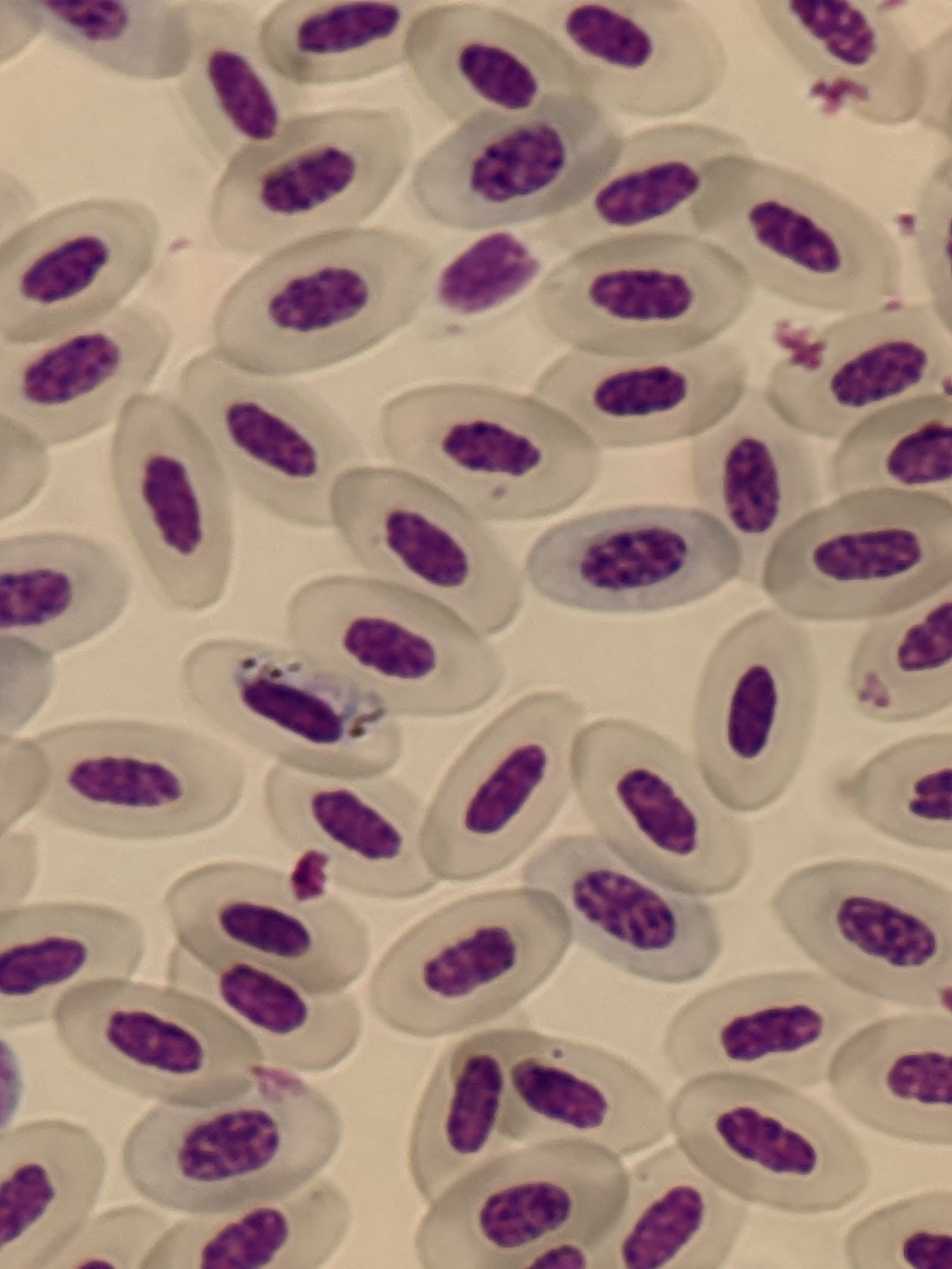
**

***Leucocytozoon* parasite**

***Haemoproteus* parasite**
